# Consequences of Hyponatremia: Central Pontine and Extrapontine Myelinolysis in a Chronic Alcohol User

**DOI:** 10.7759/cureus.61360

**Published:** 2024-05-30

**Authors:** Devyansh Nimodia, Pratapsingh Parihar, Shubhi Gaur, Prasad Desale, Komal Mishra

**Affiliations:** 1 Radiodiagnosis, Jawaharlal Nehru Medical College, Datta Meghe Institute of Higher Education and Research, Wardha, IND

**Keywords:** neurological complications, chronic alcohol use, hyponatremia, extrapontine myelinolysis, central pontine myelinolysis

## Abstract

Central pontine myelinolysis (CPM) and extrapontine myelinolysis (EPM) are rare neurological disorders associated with rapid correction of hyponatremia, particularly in individuals with chronic alcohol use. We present the case of a 52-year-old male with a history of chronic alcoholism who developed CPM and EPM following correction of severe hyponatremia. The patient presented with dysarthria, hemiparesis, and altered mental status, which progressed rapidly to pseudobulbar features and spastic quadriparesis. Neuroimaging revealed characteristic findings of CPM and EPM. Treatment with intravenous dexamethasone, intravenous immunoglobulin (IVIG), and methylprednisolone led to gradual neurological improvement. The patient showed significant recovery after two months, highlighting the importance of early recognition and cautious management of electrolyte disturbances in high-risk individuals to prevent devastating neurological complications.

## Introduction

Central pontine myelinolysis (CPM) and extrapontine myelinolysis (EPM) are rare neurological disorders characterized by demyelination of the central and extrapontine white matter, respectively. These conditions often occur as a consequence of rapid correction of hyponatremia, particularly in patients with chronic alcoholism [1,2]. CPM typically presents with quadriparesis, pseudobulbar palsy, and altered mental status, while EPM may manifest with a variety of neurological symptoms, including dysarthria, tremors, and ataxia [3]. The pathophysiology of CPM and EPM involves the rapid osmotic shifts in the brain's white matter following the correction of hyponatremia. This leads to demyelination and subsequent neurological deficits [4]. Previous studies have demonstrated that chronic alcohol use may predispose individuals to the development of these conditions, possibly due to alterations in osmoregulation and electrolyte balance [5].

The diagnosis of CPM and EPM is primarily based on neuroimaging findings, with magnetic resonance imaging (MRI) being the preferred modality due to its higher sensitivity in detecting demyelinating lesions [6]. Characteristic MRI findings include hyperintense lesions in the central pons for CPM and the involvement of other brain regions, such as the basal ganglia, thalamus, and cerebellum, for EPM [7,8]. Treatment of CPM and EPM remains challenging, with no consensus on the optimal management strategy. Current approaches typically involve supportive care and immunomodulatory therapy to reduce inflammation and promote remyelination [9,10]. However, the prognosis varies widely, with some patients experiencing significant neurological recovery while others may have permanent disabilities [11]. This case underscores the importance of careful monitoring and management of electrolyte disturbances, particularly in high-risk individuals such as those with chronic alcohol use, to prevent the development of CPM and EPM. Further research is needed to elucidate the underlying mechanisms and improve treatment outcomes for these rare neurological disorders.

## Case presentation

In the emergency department of our hospital, a 52-year-old male patient, known for chronic alcohol consumption, presented with complaints of speech slurring and weakness in both upper and lower limbs, persisting for the past 20 days. Over the preceding three years, he had maintained a daily alcohol intake of 360 ml. He had no significant medical history and was not on any medications. Physical examination revealed pallor and clubbing. Upon admission, the patient exhibited disorientation to time, place, and individuals. Neurological assessment disclosed dysarthria, left hemiparesis (with a power of 3/5 in both upper and lower limbs), and a right-sided plantar extensor response.

Routine investigations were initiated, including complete blood count, random blood sugar, liver and renal function tests, and serology. Laboratory analysis revealed severe hyponatremia with a serum sodium level of 105 mmol/L (normal range: 135 and 145 mEq/L), which was subsequently corrected to 124 mmol/L after 24 hours and 132 mmol/L after 48 hours through vigorous fluid resuscitation with Ringer's lactate and 0.5% NS IV solutions. The patient exhibited residual confusion, emotional lability, and perseveration despite improved hydration status. By the seventh day of admission, his clinical condition deteriorated with exacerbation of confusion, reduced verbal output, and decreased physical activity. On the eighth day, characteristic pseudobulbar features such as dysarthria, dysphagia, facial diplegia, and emotional lability emerged, accompanied by mild spastic quadriparesis.

Progressing rapidly, by the twelfth day, the patient manifested a nearly complete "locked-in" syndrome characterized by facial diplegia, severe spastic quadriparesis, and inability to vocalize or swallow, yet retained visual tracking abilities in all directions. Cerebrospinal fluid analysis revealed an elevated protein level of 53 mg/dl (normal range: 15-45 mg/dl) with no evidence of other etiological factors such as hypoxia, prolonged hypotension, hypoglycemia, or toxic exposures.

**Figure 1:**
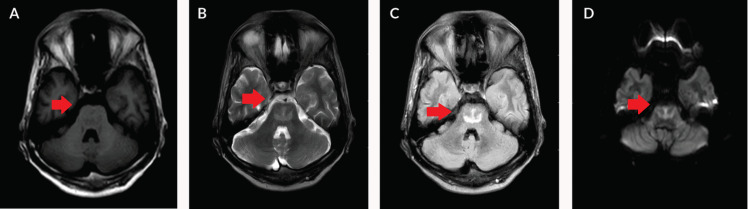
Evidence of altered signal intensity lesion noted in the pons appearing hypointense on T1WI (A), hyperintense on T2WI/FLAIR (B, C), showing diffusion restriction on DWI (D) in the axial section FLAIR: fluid-attenuated inversion recovery; DWI: diffusion-weighted imaging

Subsequent imaging studies, including a computed tomography (CT) scan on the ninth day, showed no abnormalities. However, MRI of the brain and cervical spine revealed hypointensity in the pons on axial T1-weighted images, with symmetrical hyperintense patches in the central region of the pons on axial T2-weighted images, consistent with CPM (Figure 1).

**Figure 2:**
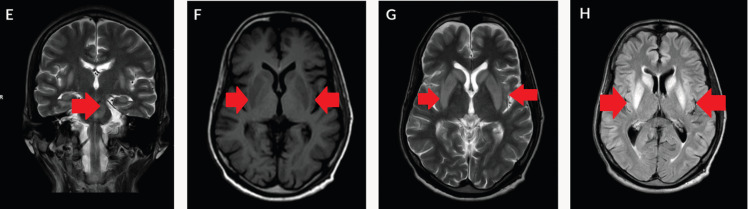
Evidence of altered signal intensity lesion in pons appearing hyperintense on coronal T2WI (E); in the bilateral caudate nucleus, lentiform nucleus, posterior limb of the internal capsule, and external capsule appearing hypointense on axial T1WI (F), hyperintense on T2WI/FLAIR (G, H) in the axial section FLAIR: fluid-attenuated inversion recovery

Additionally, altered signal intensity was observed in the bilateral caudate nucleus, lentiform nucleus, posterior limb of the internal capsule, and external capsule, suggesting EPM (Figure 2). Treatment involved intravenous administration of dexamethasone, intravenous immunoglobulin (IVIG), and methylprednisolone, resulting in gradual neurological improvement.

After 24 days of coma, the patient's neurological condition began to improve, with significant recovery noted after two months. He regained normal cognitive function, near-full motor control of facial and extremity muscles, and regained independence in activities of daily living. The cerebrospinal fluid analysis demonstrated normal intrathecal IgG production and no evidence of blood-brain barrier disruption.

## Discussion

CPM and EPM are rare neurological disorders characterized by demyelination of the central and extrapontine white matter, respectively. These conditions typically occur following the rapid correction of hyponatremia, particularly in patients with chronic alcoholism [12]. Our case highlights the devastating neurological consequences of untreated hyponatremia in a patient with a history of chronic alcohol use. The pathophysiology of CPM and EPM involves the rapid osmotic shifts in the brain's white matter following the correction of hyponatremia. This leads to the destruction of myelin sheaths and subsequent neurological deficits [4]. The severity of the neurological manifestations correlates with the extent of demyelination and neuronal damage.

Diagnosis of CPM and EPM relies on neuroimaging, with MRI being the preferred modality due to its high sensitivity in detecting characteristic lesions [13]. In our case, MRI revealed symmetrical hyperintense patches in the central region of the pons on T2-weighted images, consistent with CPM and alterations in signal intensity in extrapontine regions, indicative of EPM. Treatment of CPM and EPM primarily involves supportive care and immunomodulatory therapy to halt the progression of demyelination and promote neurological recovery [14]. In our case, the patient received intravenous dexamethasone, IVIG, and methylprednisolone, which gradually improved neurological symptoms.

The long-term prognosis of CPM and EPM varies widely, with some patients experiencing partial or complete recovery, while others may be left with permanent neurological deficits [15]. Fortunately, our patient showed significant neurological improvement after two months, with near-complete resolution of symptoms. This case underscores the importance of cautious monitoring and management of electrolyte disturbances, particularly in high-risk individuals such as those with chronic alcohol use. Early recognition and appropriate treatment of hyponatremia can prevent the development of devastating neurological complications associated with CPM and EPM.

## Conclusions

In conclusion, our case highlights the significant neurological consequences of CPM with EPM in a patient with chronic alcohol use and untreated hyponatremia. Through careful monitoring and management of electrolyte disturbances, particularly in high-risk individuals, such devastating neurological complications can be mitigated. Early recognition, appropriate diagnostic imaging with MRI, and timely initiation of immunomodulatory therapy are essential for optimizing patient outcomes. While the prognosis of CPM and EPM can vary, our patient demonstrated substantial neurological improvement after treatment, emphasizing the importance of prompt intervention in mitigating long-term sequelae. Further research is warranted to elucidate optimal treatment strategies and long-term outcomes in similar cases.
